# Intonational production as a window into children’s early pragmatic competence: The case of the Norwegian polarity focus and two *jo* particles

**DOI:** 10.3389/fpsyg.2023.1116842

**Published:** 2023-04-11

**Authors:** Line Sjøtun Helganger, Ingrid Lossius Falkum

**Affiliations:** ^1^Department of Languages and Literature Studies, University of South-Eastern Norway, Kongsberg, Norway; ^2^Department of Philosophy, Classics, History of Arts and Ideas, University of Oslo, Oslo, Norway; ^3^Department of Linguistics and Scandinavian Studies, University of Oslo, Oslo, Norway

**Keywords:** pragmatic development, intonation, polarity focus, response particles, pragmatic particles, metarepresentation, epistemic vigilance, relevance theory

## Abstract

The use of the Norwegian intonation pattern Polarity Focus highlights the polarity of a contextually given thought and enables the speaker to signal whether she believes it to be a true or false description of some state of affairs. In this study, we investigate whether preschool children can produce this intonation pattern and what their productions reveal about the development of their early pragmatic abilities. We also explore their use of Polarity Focus in combination with two particles encoded by the linguistic form *jo*: a sentence-initial response particle, and a sentence-internal pragmatic particle. We used a semi-structured elicitation task consisting of four test conditions of increasing complexity to shed light on the developmental trajectory of the mastery of Polarity Focus. Our results show that already from the age of 2 children are proficient users of this intonation pattern, which occurs in three out of four conditions for this age group. As expected, only 4- and 5-year-olds produced Polarity Focus in the most complex test condition that required the attribution of a false belief. We further found production of sentence-initial response particle *jo* by all age groups, both in combination with Polarity Focus and alone. Production of the sentence-internal pragmatic particle *jo*, felicitously co-occurring with Polarity Focus, emerges around age 3. This study presents the first experimental evidence of Norwegian children’s mastery of intonation as a communicative device in language production and their use of the two *jo* particles. We show how intonational production can be used as a window into children’s early pragmatic competence: The mastery of the production of Polarity Focus can be seen as an early linguistic manifestation of the cognitive abilities for the attribution of thoughts and epistemic vigilance towards propositional content.

## 1. Introduction

Imagine that you are talking to your three-year-old who is trying to explain that he sent a letter to his buddy at the kindergarten earlier that day. To make sure that you have understood what he has just told you, you say: *Så han var* *ikke i barnehagen?* (‘So, he was not at kindergarten?’). The child replies as in (1) below:^1^

(1) Å (3;10): ((han(^1^VAR-i_AP_)_IP_)(^2^barnehagen_AP_)_IU_)
        L* H^−^      H*L      (H)   L%
             he WAS-in kindergarten_DEF_
             ‘He was at kindergarten (despite what you are saying).’

In (1), the child provides the information that his friend was present at the kindergarten that day. However, the child also signals that there is an opposition between what you seem to think (that his friend was not present at the kindergarten) and the actual state of affairs (that his friend was present at the kindergarten). The child denies the truth of the proposition expressed by your utterance, and he does so by using a particular intonation pattern, the so-called Polarity Focus (PF; e.g., Fretheim, 2002), characterized by a focal accentuation of a polarity carrier (in (1), the finite verb *var* (‘was’)) followed by an additional accentuation later in the utterance (in (1), *barnehagen* (‘the kindergarten’)). What is particular about such PF utterances compared to utterances realized without PF, is that the use of PF signals that the only new information in the utterance is the truth value of the proposition expressed by the speaker. This makes PF a valuable tool for a speaker who wants to convince her interlocutor of whether a contextually given thought is a true or false description of some state of affairs.

For a speaker to be able to signal a denial of her interlocutor’s thought, it must be manifest to her that her interlocutor believes this thought, that is, she must be capable of mentally representing that her interlocutor believes this thought and accepting this as true or probably true (Sperber and Wilson, 1986/1995, p. 39; Wilson, 2012). The use of PF therefore requires the abilities to metarepresent and attribute thoughts. Also, since the production of PF arguably requires the speaker to be attentive to, evaluate, and express an attitude (of endorsement or denial) toward the truth-conditional content of an attributed thought, it is a higher-level metarepresentational ability that is required (*cf.* Wilson, 2012), in addition to a capacity for ‘epistemic vigilance’ toward utterance content (*cf.* Sperber et al., 2010). The example in (1) above (taken from the first author’s diary notes of her son) suggests that PF may occur early in children’s language production. In this study, we ask whether preschool children tend to produce this intonation pattern, and, if so, what their production of PF utterances can reveal about the development of their early pragmatic abilities.

Instead of (1), the child could also have had responded as in (2a), (2b) or (2c) below to communicate the same (or similar) content:

(2) a. A: *Så han* *var* *ikke i barnehagen?*
        ‘So, he was not at the kindergarten?’
      B1: (((^1^JO_AP_)_IP_)_IU_)
      Yes_RESP.PART_
       ‘Yes (contrary to what you are saying, he was at the kindergarten).’
     b. B2: (((^1^JO_AP_)_IP_)_IU_), ((han(^1^VAR-i_AP_)_IP_)(^1^barnehagen_AP_)_IP_)_IU_)
      Yes_RESP.PART_ he WAS-in kindergarten_DEF_
       ‘Yes, he was at the kindergarten (despite what you seem to think).’
       c. B3: ((han(^1^VAR-jo-i_AP_)_IP_)(^1^barnehagen_AP_)_IU_)
      he WAS-_PART_-in kindergarten_DEF_
     ‘You know, he was at kindergarten (despite what you seem to think).’

In addition to investigating PF production, this study explores children’s use of two particles, both orthographically expressed as *jo*, which often co-occur with PF and share some of its pragmatic features: they both enable the speaker to signal her attitude toward the truth value of the proposition expressed. *Jo* can appear either as a sentence-initial response particle (Fretheim, 2017), such as in (2ab) above, or as a sentence-internal pragmatic particle (Berthelin and Borthen, 2019), as in (2c).^2^

According to Fretheim (2017), the sentence-initial response particle *jo* is used to contradict a communicated negation by affirming the embedded positive proposition. Thus, both PF and the response particle *jo* require that the speaker metarepresents a contextually manifest thought and they both relate to the truth of the proposition expressed.^3^ The response particle *jo* can be used as a single word response, such as in (2a) above, or it can precede a PF utterance, such as in (2b).

As to the sentence-internal pragmatic particle *jo*, Berthelin and Borthen (2019) suggest that its semantic contribution is to signal that the proposition expressed should be interpreted as mutually manifest to the speaker and hearer, and to signal that the utterance can be taken as a premise for deriving a contextual implication. Sentence-internal *jo* therefore naturally accompanies PF in (2c), providing the hearer with an additional cue to the speaker’s intention: that the hearer should accept as true that the speaker’s friend was indeed present at the kindergarten. Because of the overlapping pragmatic features between PF and the *jo* particles described above, more careful analyses of these early *jo* productions may serve to cast light on the development of metarepresentational abilities in children.

In (1) above, PF was used to signal the speaker’s denial of the truth of the metarepresented thought. However, PF can also be used to affirm the truth of a thought, as in (3) below where A concludes after hearing that B’s fried was present at kindergarten when B had sent his letter:

(3)  A: *Da fikk han brevet med en gang.*
    ‘Then he received the letter right away.’
  B: (((^1^han_AP_)(^1^FIKK_AP_)_IP_)(^1^brevet-med_AP_)(^1^en_AP_)(^1^gang_AP_)_IU_)
    L*(H)    L* H^−^     L*    (H)    L*(H) L*(H) L%
      He RECEIVED letter_DEF_-with one time
     ‘He DID receive the letter right away (just as you seem to think)!’

The PF in B’s utterance is realized as a focal accentuation of the finite verb *fikk* (‘received’) followed by three additional non-focal accentuations. Using PF, B metarepresents and affirms the truth of the proposition explicitly expressed by A in (3).

PF can also be used to affirm or deny a thought that is not explicitly mentioned but attributed to someone (or to oneself). Consider the conversation in (4) below:

(4) A: *Jeg kommer meg ikke til butikken!*
       I come me not to grocery store_DEF_
       ‘I can’t get to the grocery store!’
     B (who knows A has an electric car):   (((^1^bilen_AP_)(^1^ER_AP_)_IP_)((^2^LADET_AP_)_IP_)_IU_)
  L*(H)     L*H^−^     H*L     H-H%
  car_DEF_ IS CHARGED

‘The car is charged (despite what you seem to think)’.

Here B’s utterance is realized with PF, involving a focal accentuation of the finite verb *er* (‘is’) followed by another (focal) accentuation of the infinite verb form *ladet* (‘charged’). A expresses that he cannot get to the grocery store, and by responding with a PF utterance, B communicates that she denies a (false) belief that she attributes to A: that he cannot use his (electric) car to drive to the grocery store because it is discharged.

The analysis of the use and function of PF has an affinity with the Relevance Theoretic notion of ‘echoic utterances’. Using an echoic utterance, the speaker metarepresents and attributes a thought or utterance with a similar content to someone else (or to the speaker herself), and at the same time conveys the speaker’s attitude to this thought or utterance. It is this signaling of the speaker’s attitude to the attributed thought, which is characteristic of echoic utterances, and by which they achieve relevance (Wilson, 2012, p. 249). Using an echoic utterance is one linguistic tool available to the speaker if she intends to modify her interlocutor’s epistemic state. Utterances carrying PF serve a similar communicative function in that they involve the affirmation or denial of an attributed proposition as a description of some state of affairs, and could in this way be said to involve an echoic element.

An overarching aim of this study is to gain a deeper understanding of intonation as part of a broader pragmatic competence. According to Relevance Theory (Sperber and Wilson, 1986/1995), intonation serves an important pragmatic function by contributing to an utterance’s relevance (where ‘relevance’ is understood as a trade-off between so-called cognitive effects and processing effort). On this view, intonation functions procedurally as a guide to the speaker’s intended interpretation:^4^ by signaling an utterance’s information structure it contributes to making some contextual implications more salient than others (Fretheim, 2002; Wilson and Wharton, 2006; Wilson, 2011; Scott, 2021).

The early emergence of prosodic competence in first language acquisition (for an overview, see Kehoe (2013)) and the pragmatic nature of intonation provide us with an opportunity to use intonation as a window into children’s early pragmatic competence. Children’s acquisition of intonation in the period prior to five years of age is still a quite unexplored field of research (Peppé and Wells, 2014). Furthermore, although there are studies on the role of (intonational) focus in pragmatic reasoning (e.g., Tomlinson et al., 2017; Gotzner and Spalek, 2019), there have been few attempts to combine suprasegmental phonology with cognitive pragmatic theory in the study of language acquisition (Wharton, 2012, 2020). Thus, the question of how children’s ability to master intonation as a communicative device develops remains largely unresolved. Analyses of children’s production of intonation utterances can provide us with a deeper understanding of what this ability amounts to. In addition, this study presents the first attempt of accounting for Norwegian speaking children’s use of intonation as a communicative device in language production.

### 1.1. Previous research

While some developmental studies have investigated children’s ability to produce intonational focus (e.g., Wieman, 1976; Wells et al., 2004; Romøren and Chen, 2021), we know of no previous studies that have specifically investigated children’s production of PF. However, the literature discusses a similar intonation pattern, often referred to as ‘Verum Focus’ (e.g., *The house ISn’t on fire*; Gussenhoven, 1983, p. 406). In an elicitation experiment, Turco et al. (2014) showed adult participants pictures of different situations (e.g., a man washing a car). Participants then heard prerecorded utterances where the depicted situations were negated (e.g., *The man is NOT washing the car*). Together, the visual and audio stimuli served as a context for eliciting Verum Focus utterances, where the truth value of the negation provided in the audio stimuli was to be corrected (e.g., *The man IS washing the car*). Results showed that that German adult speakers produced Verum Focus in more than 70% of the cases in these ‘polarity correction’ contexts.^5^

In an adaptation of Turco et al.’ (2014) study, Dimroth et al. (2018) investigated German four-to six-year-olds’ production of this intonation pattern but found only a small number of occurrences (5 out of 175 trials). In their adult control group, Verum Focus occurred in 53 out of 99 trials. However, despite several similarities between Verum Focus and PF, the two notions are not equivalent: Verum Focus is used in a broader sense, also including contexts where the polarity of a metarepresented proposition is not really at question, such as the ‘polarity contrast’ context of Dimroth et al. (2018). In this context, a confederate describes a picture only visible to him, using a negative utterance (e.g., *In my picture the child is not eating the candies*) and the participant’s task is to respond by describing her own picture. This picture shows the affirmative version of the confederate’s picture (e.g., a child eating candies), only accessible to the participant herself, leading to responses such as *On mine the child HAS eaten the candies* (Dimroth et al., 2018, p. 276). In this context, the accentuation of the finite verb does not highlight the polarity of any proposition; the issue is not whether or not it is true that the child in the participant’s picture eats candies. Rather the participant’s response highlights the difference (contrast) between the motive in the participant’s and the confederate’s pictures.

As to the Norwegian *jo* particles, they have not previously been studied in a developmental context. Noveck et al. (2021) investigated children’s production of the French equivalent to the Norwegian sentence-initial response particle *jo*, the response particle *si*. They describe the response particle as “a pragmatically rich response that addresses the questioner’s epistemic state” (*ibid.*, p. 4). *Si* can be used to respond affirmatively when a negative question at the surface structure turns out to be a false negative one, implicitly signaling the questioner’s positive belief (e.g., *It is not in the white box?*). In Noveck and colleagues’ study, participants answered a question of whether a candy was in a box or not. Each trial started with a puppet declaring his prior belief about the candy’s whereabouts before the participant inspected the box. Then the puppet asked either an affirmative question (e.g., *It is in the white box?*) or a negative question (e.g., *It is not in the white box?*). Crucially, in the *si*-eliciting condition the puppet asked a negative question, but the box contained the candy.

The results showed that six-year-olds are adult-like in their uses of *si* but four-year-olds are not. Although the four-year-olds showed adult-like accuracy rates (where accuracy was understood in terms of pragmatically felicitous responses), answering *Oui*, *Non* and *Si* to the puppet’s question just as correctly as the six-year-olds and adults did, they were strikingly faster than the six-year-olds and adults in responding *si* in the context of a false negative question. According to Noveck et al. (2021) this accurate, but unexpectedly fast response indicates that four-year-olds rely on a minimal semantic representation of *si* when answering the question (in rejecting the content of the false-negative question), but do not yet fully appreciate its pragmatic complexity which involves “[modifying] the questioner’s epistemic state so that it aligns better with the answerer’s” (*ibid.*, p. 22).

If Noveck et al. (2021) are right in their analysis of the four-year-olds’ pragmatic immaturity–and assuming that the response particles *si*, *jo* and PF broadly serve the same pragmatic function of metarepresenting a contextually manifest thought and expressing an attitude toward the truth-conditional content of this thought–we should not expect four-year-olds, and certainly not children younger than four years of age, to produce PF.

However, we are not entirely convinced by the conclusion Noveck et al. (2021) draw regarding four-year-olds’ pragmatically limited use of *si*. From the developmental literature, we know that already around the age of two, children have a capacity for metarepresentation (Leslie, 1987), they can reject false and accept true statements (Lyon et al., 2013), and they can spontaneously contradict and correct assertions that they believe to be false (Pea, 1982). Furthermore, already from around 14 months of age, children’s perspective-taking abilities include the understanding that attitudes of others to objects of joint attention may differ from their own (O’Madagain and Tomasello, 2021). It seems puzzling to us that they would not also make use of these abilities when producing *si* in appropriate contexts. Arguably, the main informative intention of a speaker who uses this particle is to convey her denial of a metarepresented thought (why else would she use it?). Furthermore, it is likely that her goal in conveying this is to modify her interlocutor’s epistemic state. This would seem to involve an understanding that goes beyond accessing the minimal semantic representation (i.e., the mere rejection of a negative surface structure), and which includes beginning mastery of the pragmatic processes involved in the mature use of the utterance to affirm the questioner’s positive belief. It is this pragmatically rich understanding that seems to be involved in the use of utterances containing the response particle *jo*, PF, or a combination of the two, such as in (2b) above.

### 1.2. Hypotheses

We hypothesize that preschool children should be able to produce PF in appropriate contexts. This hypothesis is based partly on anecdotal observations of children’s early PF productions from diary notes and private recordings, and partly on what we know about the early development of some of the prerequisite abilities for use of PF (*cf.* Pea, 1982; Leslie, 1987; Lyon et al., 2013; O’Madagain and Tomasello, 2021), as well as children’s pragmatic sophistication in related domains such as the ability to draw scalar implicatures (Pouscoulous et al., 2007), to grasp presuppositions (Berger and Höhle, 2012), and to appropriately use referring expressions (Matthews et al., 2006). However, given that the ability to linguistically express an understanding of false beliefs appears around children’s fourth birthday (Wellman et al., 2001), we would only expect children aged four years and older to produce PF in the most complex context where they have to infer and attribute a false (or ignorant) belief to their interlocutor (*cf.* (4) above). The study’s hypotheses are preregistered in OSF: https://osf.io/3asu5/.

In the examples in (1)–(3) above, the proposition echoed is explicitly expressed by the interlocutor prior to the speaker’s PF utterance and is therefore easily accessible. We hypothesize that the use of PF in such contexts is acquired earlier than in contexts where the proposition echoed must be inferred (such as in (4) above).

Findings from the developmental literature suggest that the presence of negation increases the complexity of utterances (Just and Carpenter, 1971; Clark and Chase, 1972). We hypothesize that use of PF to affirm a positive proposition is acquired earlier than the denial of a positive proposition, followed by the ability to deny a prior negative belief.

We expect the earliest starting point of PF production to be around two years of age, by the age typically developing children have usually started to produce word combinations (Kristoffersen and Simonsen, 2012). This hypothesis is based on the intonational criteria for PF production: An utterance realized with two accentuations must consist of at least two words (Fretheim and Nilsen, 1993). Furthermore, Lyon et al. (2013) have shown that children are able to accept and reject true and false statements before their second birthday, suggesting a developing ability for epistemic vigilance toward utterance content (Sperber et al., 2010).

## 2. Method

### 2.1. Participants

This study includes 92 children within the age range of 2;2 to 5;9 years, divided into four groups: two-year-olds (*n* = 20), three-year-olds (*n* = 20), four-year-olds (*n* = 31), and five-year-olds (*n* = 21). Seven additional participants were omitted from the analyses because they produced no comprehensible multiword utterances during the recording sessions (*n* = 6) or failed to concentrate on the experimental tasks (*n* = 1). The participants had South-East Norwegian as their first language^6^ and were recruited through kindergartens in the South-Eastern region of Norway. Prior to data collection the study received ethical approval from NSD–Norwegian Center for Research Data (project number 60923) and written parental consent was obtained. Participants were tested individually in a quiet room in the kindergarten or in their private home. To capture the intonational production of the children and as much of the context as possible, the participants were video recorded using a Sony video camera recorder HDR-CX410 with a 5.1ch surround microphone. Each session lasted for approximately 10 min.

### 2.2. Procedure and materials

Our semi-structured design involves an elicitation task combined with intermediate sections of spontaneous speech. The sections of spontaneous speech are included to make the experimental setting as similar as possible to a natural conversation. The initial unstructured conversation is especially important for establishing a relation between the participant and the experimenter, and for the participant to get acquainted with the handpuppet used in the elicitation task. It also serves to establish the relevant context for the elicitation task that follows.

First, an experimenter and a handpuppet show the participant some of the handpuppet’s toys (three rubber ducks and a little ball) during an unstructured conversation, where the handpuppet demonstrates that he is a bit forgetful. The experimenter, the handpuppet and the participant play with and talk about the toys, commenting on how they look, what they can be used for, and so on. The handpuppet explicitly states that he loves playing with his rubber ducks. In the remainder of the unstructured sections, the participant, handpuppet and experimenter talk about topics related to the structured elicitation task they go through.

Second, participants are presented with the structured PF elicitation task. Inspired by the ‘polarity correction’ context of Turco et al. (2014), we used still-life pictures as visual stimuli in three of the conditions. From Noveck et al. (2021) we adapted the procedure whereby a puppet initially explicitly states his (positive or negative) prior belief in the form of a declaration regarding some state of affairs (e.g., *I believe that the boy is eating strawberries*) before the visual stimuli is presented. Depending on the condition, the prior belief is either a match or a mismatch as a description of the picture’s motive. The crucial task for the participant is to produce a target utterance in response to the puppet’s utterance about the motive in the picture. Before the picture is presented the puppet hides so he cannot see, making it more likely that the participant will produce an utterance. If the participant does not produce any utterance, the puppet, still hiding, will ask an elicitation question to prompt the child to produce an answer (e.g., *Does the boy eat strawberries?*). Participants are not given any kind of instructions for what or how to respond, it is their spontaneous production that is of interest.

In one of the conditions, instead of expressing a belief about the content of a picture the handpuppet expresses a desire (*I wish I had something to play with while taking a bath*), suggesting that he does not remember his rubber ducks (i.e., the ones they had played with in the initial conversation). Here the production of PF is only relevant as a response if the participant has drawn the necessary inferences about a (false) belief of the handpuppet (i.e., that he does not have rubber ducks).

To familiarize the children with the procedure of responding to the puppet’s prior beliefs about the pictures, we included a familiarization trial. The four test conditions are thought to be of increasing complexity,^7^ starting with the “Positive-Affirmation” condition as the simplest one where the child only has to affirm a positive proposition. Next is the “Positive-Denial” condition where a positive proposition must be denied. Third is the “Negative-Denial” condition which involves the contradiction of a negated proposition. Fourth, and with highest complexity, is the “Inferred Belief-Denial” condition where an inferred (negative) belief must be contradicted. This increasing complexity enables the study of a potential developmental trajectory of the mastery of PF. In addition we included a Control condition, where use of PF is not relevant because the context provides no proposition of which to highlight the polarity. All participants were tested in all conditions and each participant saw a total of five test items.

The conditions were pseudo-randomized. The Inferred Belief-Denial condition was set as the third trial across participants. We did this to ensure that (i) it did not occur too soon after the visual stimuli had been presented, (ii) that retrieving the visual stimuli from memory was not too effortful, and (iii) the memory demands were the same for all children. The remainder of the conditions were randomized. See (5)–(10) below for an overview of the study’s familiarization trial and conditions:

(5) **Familiarization trial**
  Introduction by experimenter: *Now you will see a picture*.
  Prior belief (opinion) by puppet: *I love to watch pictures!*
  Visual stimuli: A sleeping dog.
  Elicitation question: *What do you see in the picture?*
(6) **Positive-Affirmation condition (Pos-Aff)**
  Introduction by experimenter: *Next is a picture of a boy*.
  Prior POSITIVE belief: *I believe that the boy is eating strawberries*.
  Visual stimuli: Match–a boy eating strawberries.
  Elicitation question: *Does the boy eat strawberries?*
  Potential PF response: (((^1^gutten_AP_)(^1^SPISER_AP_)_IP_)(^1^jordbær_AP_)_IU_)
  boy_DEF_ EATs strawberries
  ‘The boy DOES eat strawberries.’
(7) **Positive-Denial condition (Pos-Den)**
  Introduction by experimenter: *Next is a picture of a girl*.
  Prior POSITIVE belief: *I believe that the girl is throwing a ball*.
  Visual stimuli: Mismatch–a girl lying in the grass (witout any ball).
  Elicitation question: *Does the girl throw a ball?*
  Potential PF response: (((^2^jenta_AP_)(^2^KASTER-ikke_AP_)_IP_)(^1^ball_AP_)_IU_)
  girl_DEF_ THROWS-not ball
  ‘The girl does NOT throw (a) ball.’
(8) **Negative-Denial condition (Neg-Den)**
  Introduction by experimenter: *Next is a picture of a boy*.
  Prior NEGATIVE belief: *I believe that the boy is not reading a book*.
  Visual stimuli: Mismatch – a boy reading a book.
  Elicitation question: *Does the boy not read a book?*
  Potential PF response: (((^1^gutten_AP_)(^1^LESER_AP_)_IP_)(^1^bok_AP_)_IU_)
  boy_DEF_ READS book
  ‘The boy DOES read (a) book.’
(9) **Inferred belief-Denial condition (Inf-Bel)**
  Verbal stimuli: Puppet: *I wish I had something to play with while taking a bath!*
  (Visual stimuli: The rubber ducks used initially in the unstructured conversation)
  Potential PF response: ((du(^1^HAR_AP_)_IP_)((^2^BADEENDENE-dine_AP_)_IP_)_IU_)
  you HAVE RUBBER DUCKS_DEF_-yours
  ‘You DO have your rubber ducks.’
(10) **Control condition**
  Introduction by experimenter: *Next is a picture of a girl.*
  Prior NEUTRAL belief: *I don't know what the girl does*.
  Visual stimuli: A girl hugging her teddy bear.
  Elicitation question: *What is the girl doing?*
  Potential (non PF) response: ((hun(^2^koser_AP_)(^2^BAMSEN-sin_AP_)_IP_)_IU_)
  she hugs TEDDYBEAR_DEF_-hers‘She is hugging her teddy bear.’

## 3. Results

### 3.1. Production of Polarity Focus

The first author coded the full sample of 460 elicitations in the five conditions for productions of PF and presence of the *jo* particles. 20% of the data were second coded, obtaining a Cohen’s Kappa score of *κ* = 0.72, indicating substantial agreement. A third coder was used to decide in cases of disagreement.

**Figure 1:**
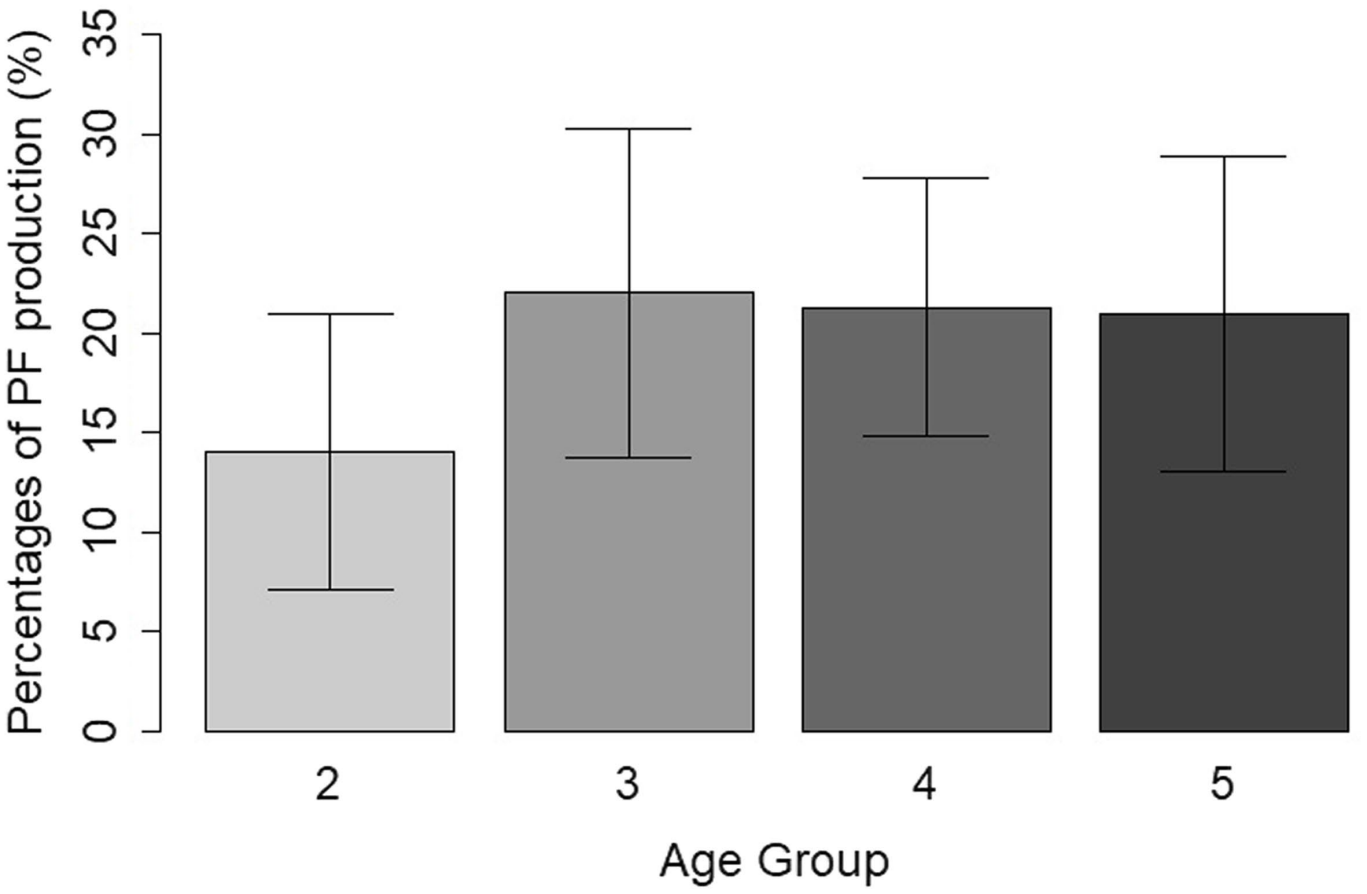
Percentages of PF productions by age group.

The results show that PF is produced by all age groups (see Figure 1). While the two-year-olds produced PF in 14% of the trials (*n* = 100), the three-year-olds produced PF in 22% of the trials (*n* = 100). The four-year-olds (*n* = 155) and five-year-olds (*n* = 105) produced PF in 21% of the trials.

To investigate the development of PF production with age, we fitted a Generalized Linear Mixed Model (GLMM) of the PF productions as a count response with an upper bound, with Age as a continuous predictor, and Subjects as a random factor using a binomial error distribution and the glmer function of the lme4 package (Bates et al., 2015) in R (version 4.2.2; R Core Team, 2022). The results show no effect of Age (*p* = 0.192). This suggests that the ability to produce PF overall is present already from the age of two years and that there is no significant increase in PF productions with age.

**Figure 2:**
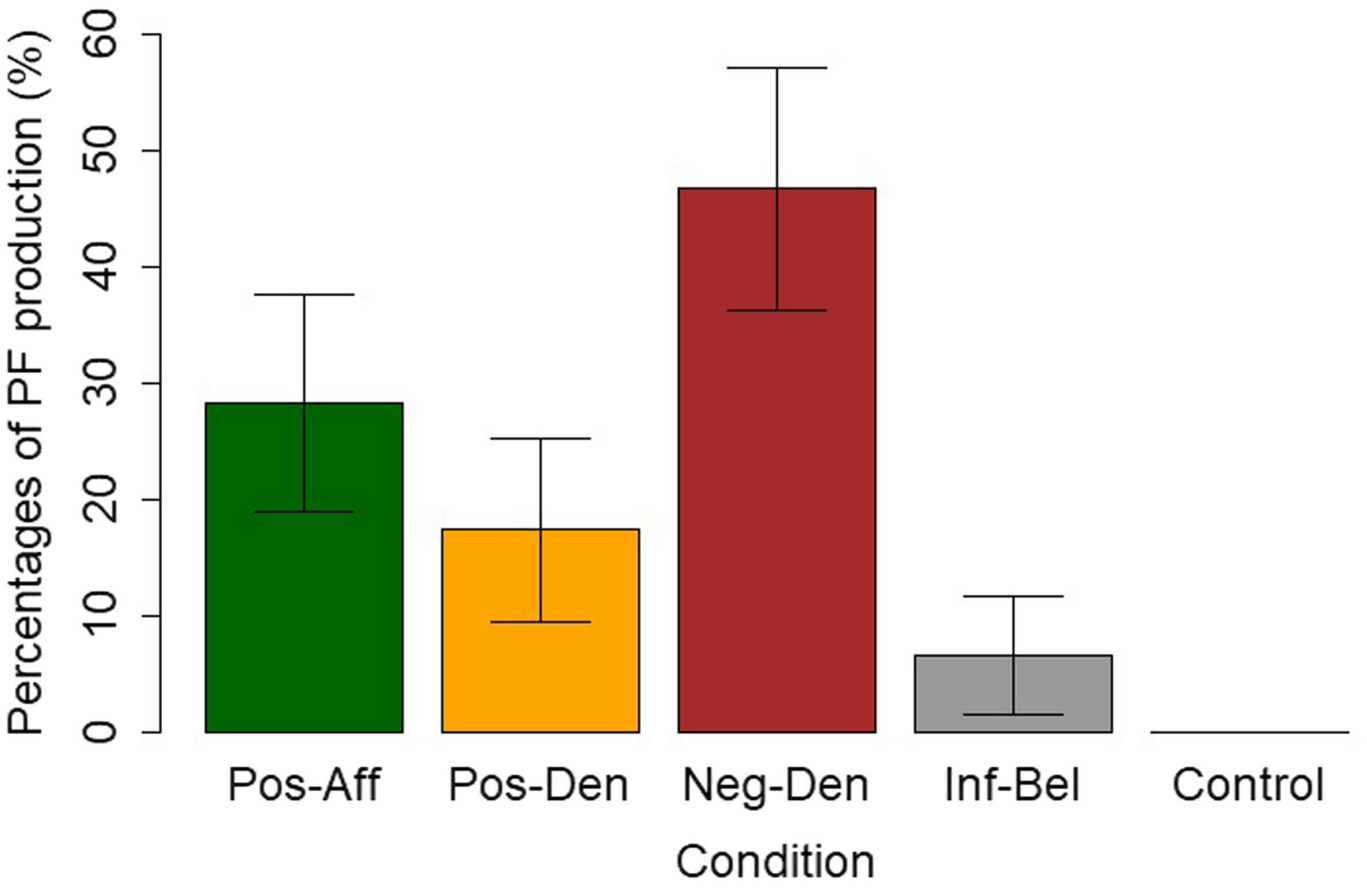
Percentages of PF productions by condition (*N* = 92).

Figure 2 shows that PF was produced in all PF conditions but the control condition, suggesting that children are using this intonation pattern in appropriate contexts. In the least complex PF condition, where a positive prior belief is affirmed by the production of PF (the Pos-Aff condition), children produced PF in 28% of the trials. In the Pos-Den condition, where the production of PF involved a contradiction of a prior positive belief, PF was produced in 17% of the trials. The Neg-Den condition, where the handpuppet’s prior belief was negative, and the production of PF involved a denial of a prior negative belief, was the one which elicited the highest number of PF with children producing this intonation pattern in 47% of the trials. In the most complex Inf-Bel condition, where the production of PF was relevant only if the participants had inferred that the handpuppet held a false belief which they then contradicted, PF was produced in 7% of the trials.

To investigate whether there are significant differences in PF productions between the four PF conditions, we fitted a GLM of the PF productions as a binomial response analyzed as a function of Condition as a categorical factor, using the glm function of the stats package in R (see Table 1 for a summary of the model). The results show that compared to the Inf-Bel condition, PF was produced significantly more often in the Pos-Aff condition (*p* = <0.001), Pos-Den condition (*p* = 0.028), and Neg-Den condition (*p* = <0.001).

**Table 1:** Summary of GLM with PF production as a binominal response analyzed as a function of condition.

	GLM of PF production ~ Condition		
Condition	Odds ratios	95% CI	*p*
(Intercept)	0.07	0.03–0.15	<0.001
Pos-Aff	5.65	2.33–15.88	<0.001
Pos-Den	3.02	1.18–8.77	0.028
Neg-Den	12.58	5.34–34.85	<0.001
Observations	368		
*R*^2^ Tjur	0.118		

**Figure 3:**
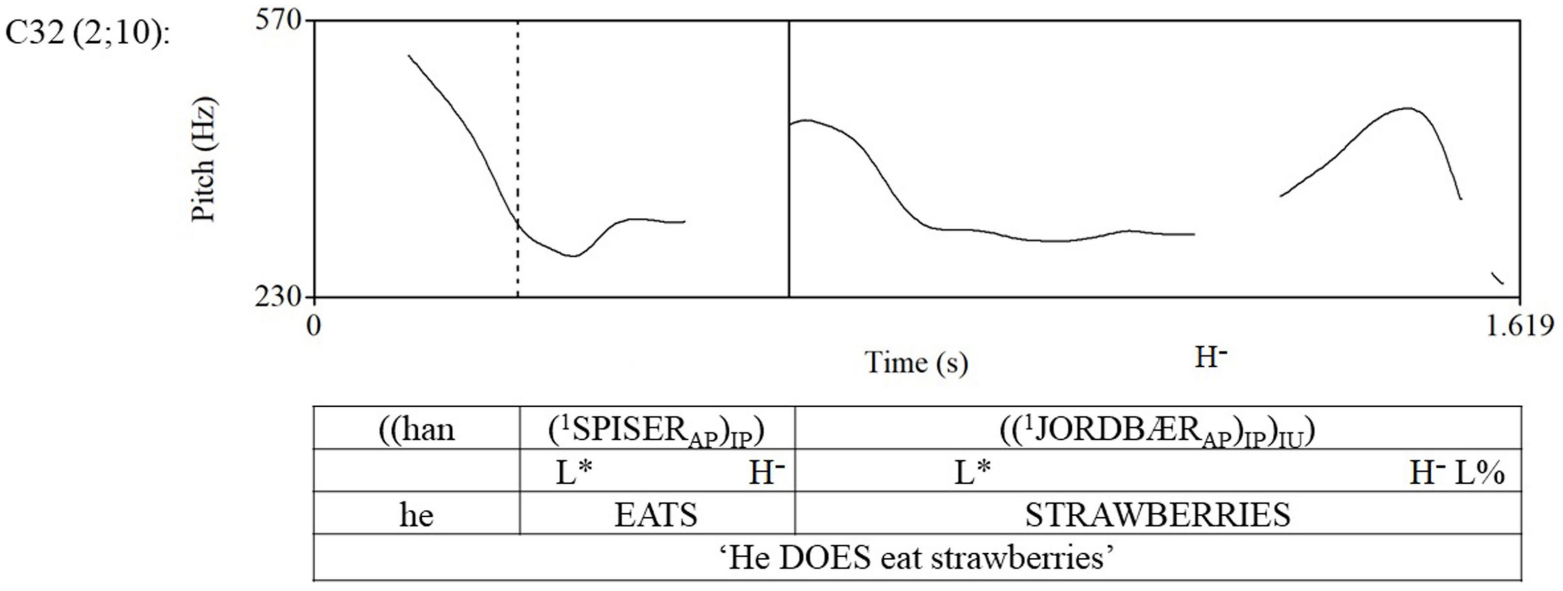
PF production by 2-year-old in the Pos-Aff condition.

**Figure 4:**
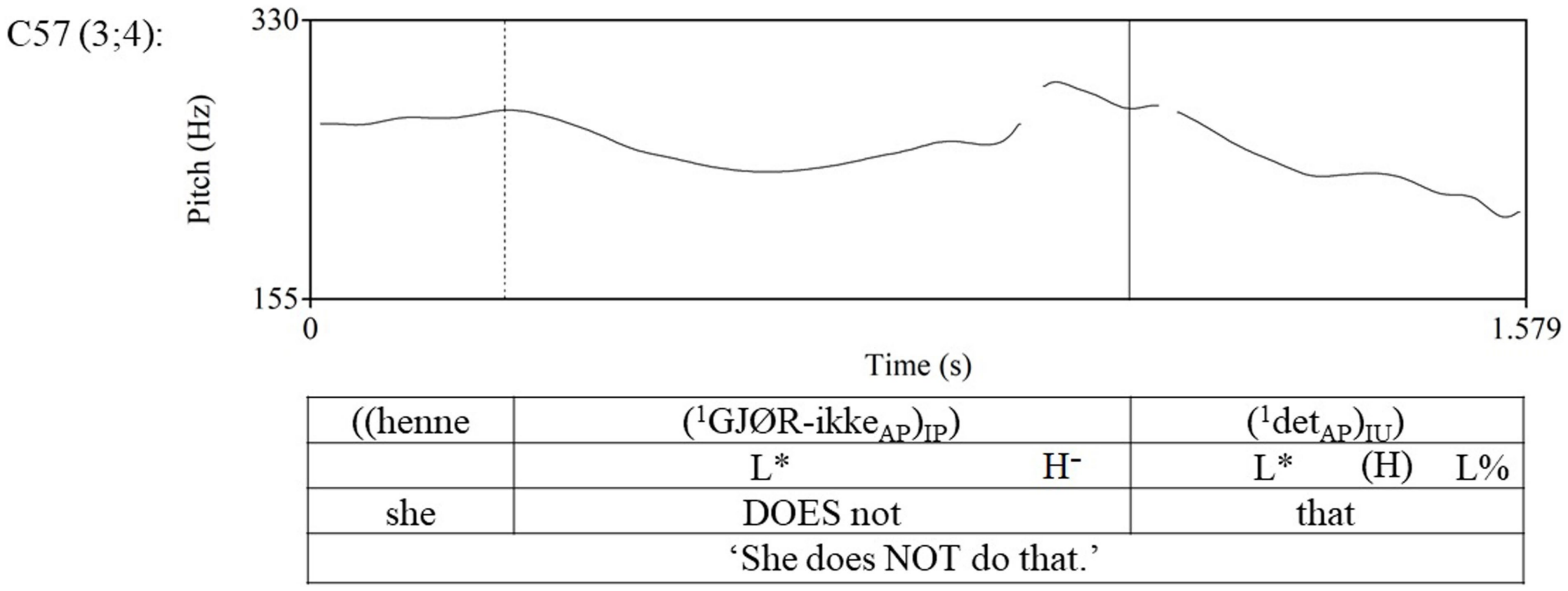
PF production by 3-year-old in the Pos-Den condition.

**Figure 5:**
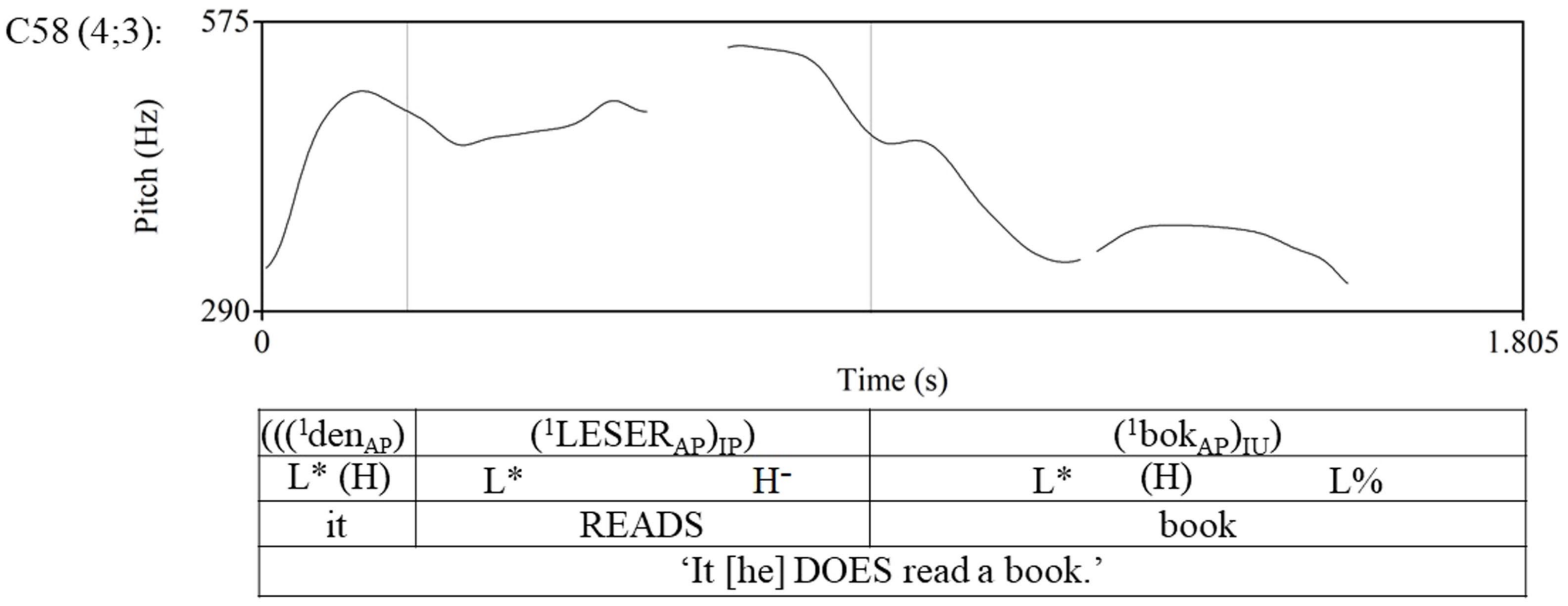
PF production by 4-year-old in the Neg-Den condition.

**Figure 6:**
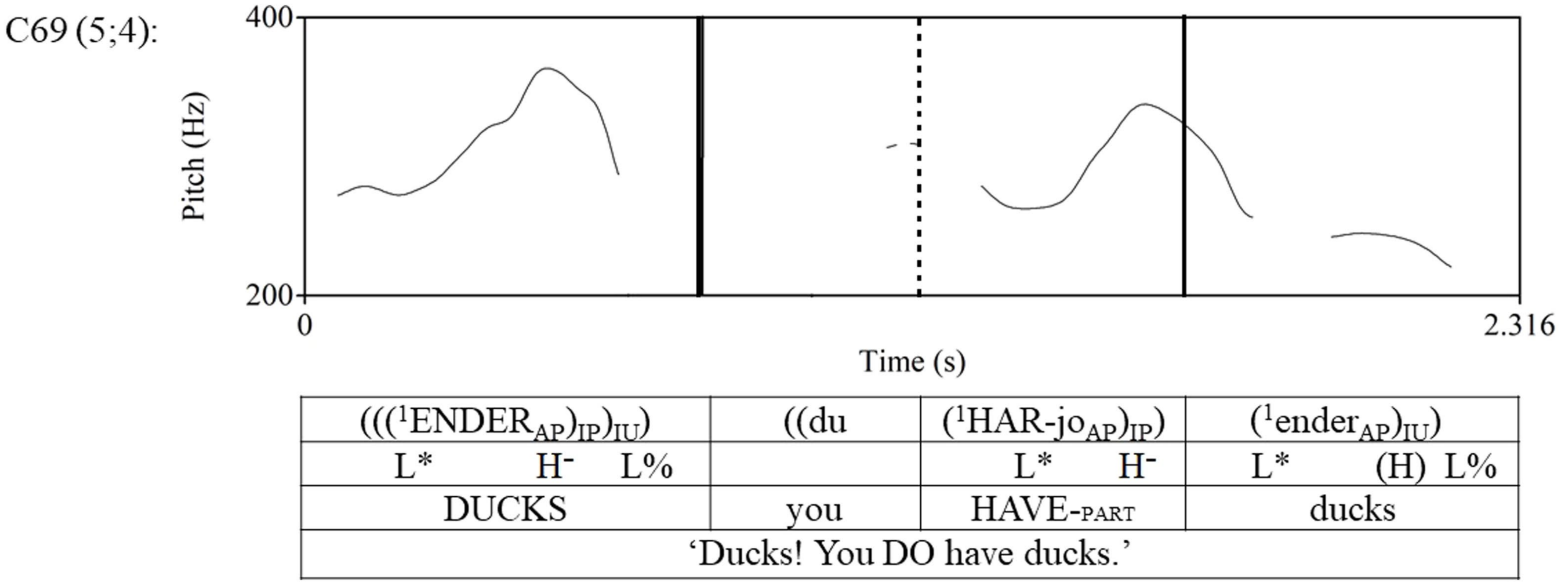
PF production by 5-year-old in the Inf-Bel condition.

Figures 3–6 below provide some examples of PF productions across conditions and age groups. Each figure consists of the fundamental frequency (F_0_) contour of the utterance and a corresponding table with four tiers. We used the software *Praat* (version 6.2.07; Boersma and Weenink, 2022) to create the F_0_-contours. The vertical lines in the F_0_-contour mark intonational boundaries that correspond to the parentheses in the transcription of the F_0_-contour given in the first tier of the table. This transcription is based on the Trondheim model (e.g., Nilsen, 1992; Fretheim, 2002) developed for analysis of Norwegian intonation. The second tier contains an annotation of the utterance’s realized tones, and the two last tiers are the English translation of the utterance. The realization of PF can be seen in the F_0_-contour as a tonal rise to an extra high tone (H^−^) on the finite verb, followed by another tonal rise, either to a high ((H)) or an extra high (H^−^) tone.

**Figure 7:**
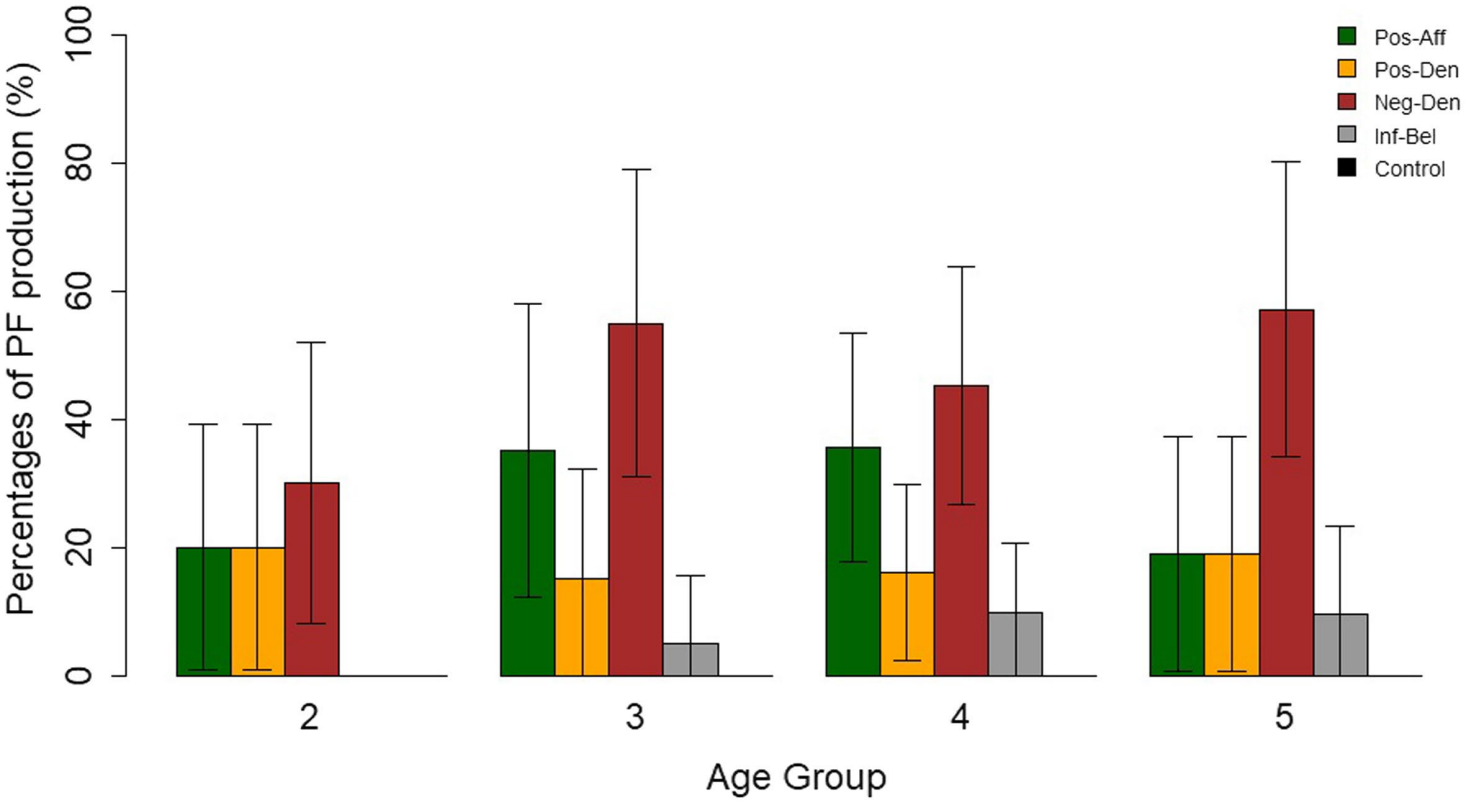
Percentages of PF productions by age group and condition.

Figure 7 below shows the PF productions by age in each condition. First, we find PF productions in three of the four PF conditions for all age groups. The Neg-Den condition has the highest percentage of PF productions for all age groups (two-year-olds: 30%; three-year-olds: 55%; four-year-olds: 45%, and five-year-olds: 57%). While the percentages of PF productions in the Pos-Den condition are quite similar across all age groups (two-year-olds: 20%; three-year-olds: 15%; four-year-olds: 16%; five-year-olds: 19%), the PF productions in the Pos-Aff condition show an equal percentage of PF productions by three-and four-year-olds (35%) and the two-and five-year-olds (20 and 19%, respectively). Furthermore, one three-year-old produced PF in the Inf-Bel condition, but most PF productions in this condition are by the two oldest age groups, although they were not frequent overall (only amounting to six occurrences in total). Taken together, these results indicate that although the overall ability to use PF is in place at the age of two, the ability to use PF in the most complex PF condition, the Inf-Bel condition, emerges later.

As shown by the error bars in Figure 7, there is great variance in the data, due to few observations in each condition when dividing responses into age groups. The models we present in what follows should therefore be interpreted with caution. To investigate any differences in performance of each age group in the four PF conditions, we fitted GLM models of PF productions as a binomial response analyzed as a function of the predictors Age Group and Condition, including their interaction, using the glm function of the stats package in R.^8^ For each model we changed reference level for Age Group (using 5-, 4-, 3- and 2-year-olds), but kept the reference level for Condition constant (using the Neg-Den condition). The results show no significant differences between the age groups. There are, however, significant differences in PF productions within the age groups: Both three-, four-, and five-year-olds produced PF significantly more often in the Neg-Den condition than in the Pos-Den condition (3-year-olds: *p* = 0.012; 4-year-olds: *p* = 0.017; 5-year-olds: *p* = 0.014) and the Inf-Bel condition (3-year-olds: *p* = 0.005; 4-year-olds: *p* = 0.004; 5-year-olds: *p* = 0.003). In addition, five-year-olds produced PF significantly more often in the Neg-Den condition than in the Pos-Aff condition (*p* = 0.014). There were no significant differences in the PF productions by the two-year-olds in the Pos-Aff, Pos-Den and Neg-Den conditions.

### 3.2. The two *jo* particles

When analyzing the production data, we noticed a striking frequency of the Norwegian word form orthographically expressed as *jo*. Although we did not aim specifically at eliciting it, this word form occurs in the participant responses in 15% (68/460) of all the trials in the structured elicitation task, and in many of the cases, it co-occurs with the participants’ PF productions. We therefore decided that the *jo* particles deserved more careful analyses. We first consider the productions of the sentence-initial response particle *jo* (47% of the occurrences), and then the productions of the sentence-internal pragmatic particle *jo* (53% of the occurrences). Given how little we know about the interaction between PF and the two *jo* particles, our analyses in this section are mainly of a descriptive and qualitative character, providing the foundation for further experimental analysis.

#### 3.2.1. Sentence-initial response particle *jo*

As Table 2 below shows, the response particle *jo* is produced most often in the Neg-Den condition (31% of the 92 Neg-Den trials). The following example shows a typical case where the response particle *jo* is followed by a PF utterance:

1. Experimenter: *Next is a picture of a boy*.

**Table 2:** Productions of sentence-initial response particle *jo* by condition.

Conditions	*Jo*
Pos-Aff	3
Pos-Den	-
Neg-Den	29
Inf-Bel	-
Control	-
Total	32

Handpuppet: *I believe that the boy is not reading a book*.

C98 (2;8): (((^1^JO_AP_)_IP_)_IU_), ((han(^1^LESER_AP_)_IP_)(^1^bok_AP_)_IU_)

YES_RESP.PART_ he READS book

‘Yes, he DOES read a book.

Table 2 shows three occurrences of the response particle *jo* in the Pos-Aff condition, a condition where *jo* should not be a relevant response since the prior belief expressed by the handpuppet is positive. One of these cases was a participant (5;1) who got excited when the handpuppet declared that he thought the boy in the upcoming picture was eating strawberries. *I don't think he is!* the participant exclaimed while waiting for the experimenter to show the picture. When the picture turned out to be a match with the handpuppet’s prior positive belief (i.e., the boy in the picture is eating strawberries), the participant contradicted his own negative belief by using the response particle *jo*, and thereby made the use of *jo* relevant.

Table 3 shows the productions of the response particle *jo* in the Neg-Den condition (*N* = 29). We see that in 72% of the trials participants responded using the response particle *jo* as a direct response to the puppet’s prior negative belief (noFNQ), that is, without the handpuppet having to ask the elicitation question. Remember that the elicitation question was asked only if the participant did not respond to the prior belief expressed by the puppet. In the remaining 28% of the trials participants responded with *jo* in the context of a false negative question (FNQ) (i.e., *Does the boy not read a book?*). Furthermore, the response particle *jo* is followed by a PF utterance in 37% of the responses. In 28% of the responses, *jo* is followed by an utterance which is not realized with PF, and in 34% of the responses, it is used as a single word response with no succeeding utterance.

**Table 3:** Productions of sentence-initial response particle *jo* in the Neg-Den condition (*N* = 29).

Neg-Den cond.	FNQ	noFNQ
Resp.part *Jo*	28%	72%
*Jo* + PF	3%	34%
*Jo* + noPF	-	28%
Simple *Jo*	24%	10%

The response in (11) above was produced by a two-year-old, but, as Table 4 shows, responses consisting of the response particle *jo* followed by a PF utterance are produced by participants in all age groups. The combination of *jo* and an utterance without PF, as well as *jo* as a single word response also occur in all age groups. In other words, sentence-initial *jo* seems to be available already from two years of age, also felicitously co-occurring with PF from this early age.

**Table 4:** Productions of the sentence-initial response particle *jo* by age group (*N* = 32).

	2-year-olds	3-year-olds	4-year-olds	5-year-olds
Resp.part *Jo*	31%	31%	22%	16%
*Jo* + PF	9%	22%	9%	6%
*Jo* + noPF	9%	3%	9%	3%
Simple *Jo*	13%	6%	3%	6%

#### 3.2.2. Sentence-internal pragmatic particle *jo*

In the utterance in Figure 6 above, repeated below as (12) for convenience, we saw an example of a PF utterance used as a response in the Inf-Bel condition. This utterance also included a sentence-internal *jo*:

1. Handpuppet: *I wish I had something to play with while taking a bath!*
  C69 (5;04): (((^1^ENDER_AP_)_IP_)_IU_), ((du(^1^HAR-jo_AP_)_IP_)(^1^ender_AP_)_IU_)
  DUCKS, you HAVE-_PART_ ducks
  ‘Ducks, you DO have ducks (remember).’

Table 5 shows the productions of sentence-internal *jo* in the five conditions. The highest number appears in the Inf-Bel condition (44%), followed by the Neg-Den condition (25%), the Pos-Aff condition (17%), the Pos-Den condition (8%), and the Control condition (6%). It further shows that sentence-internal *jo* is produced both in combination with PF and in utterances not carrying PF in all four PF conditions. The occurrences in the Control condition might seem odd, given that the use of PF is not relevant here. However, although sentence-initial *jo* naturally co-occurs with PF, addressing the polarity of a proposition expressed is not part of the semantics of this pragmatic particle. It can therefore felicitously be used in utterances produced in a neutral (or ignorant) context, such as our Control condition.

**Table 5:** Productions of the Norwegian sentence-internal pragmatic particle *jo* by condition (*N* = 36).

	Pos-Aff	Pos-Den	Neg-Den	Inf-Bel	Control
Pragm.part *Jo*	17%	8%	25%	44%	6%
PF w/*jo*	8%	6%	19%	17%	-
noPF w/*jo*	8%	3%	6%	28%	6%

Table 6 shows the productions of sentence-internal *jo* across age groups. While there are no productions among the two-year-olds, we find occurrences in the three other age groups (3-year-olds: 28%; 4-year-olds: 31%; and 5-year-olds: 42%). In addition, three-, four-, and five-year-olds produced sentence-internal *jo* both in combination with PF and in utterances without any realized PF.

**Table 6:** Productions of the Norwegian sentence-internal pragmatic particle *jo* by age group (*N* = 36).

	2-year-olds	3-year-olds	4-year-olds	5-year-olds
Pragm.part *Jo*	-	28%	31%	42%
PF w/*jo*	-	19%	17%	14%
noPF w/*jo*	-	8%	14%	28%

**Figure 8:**
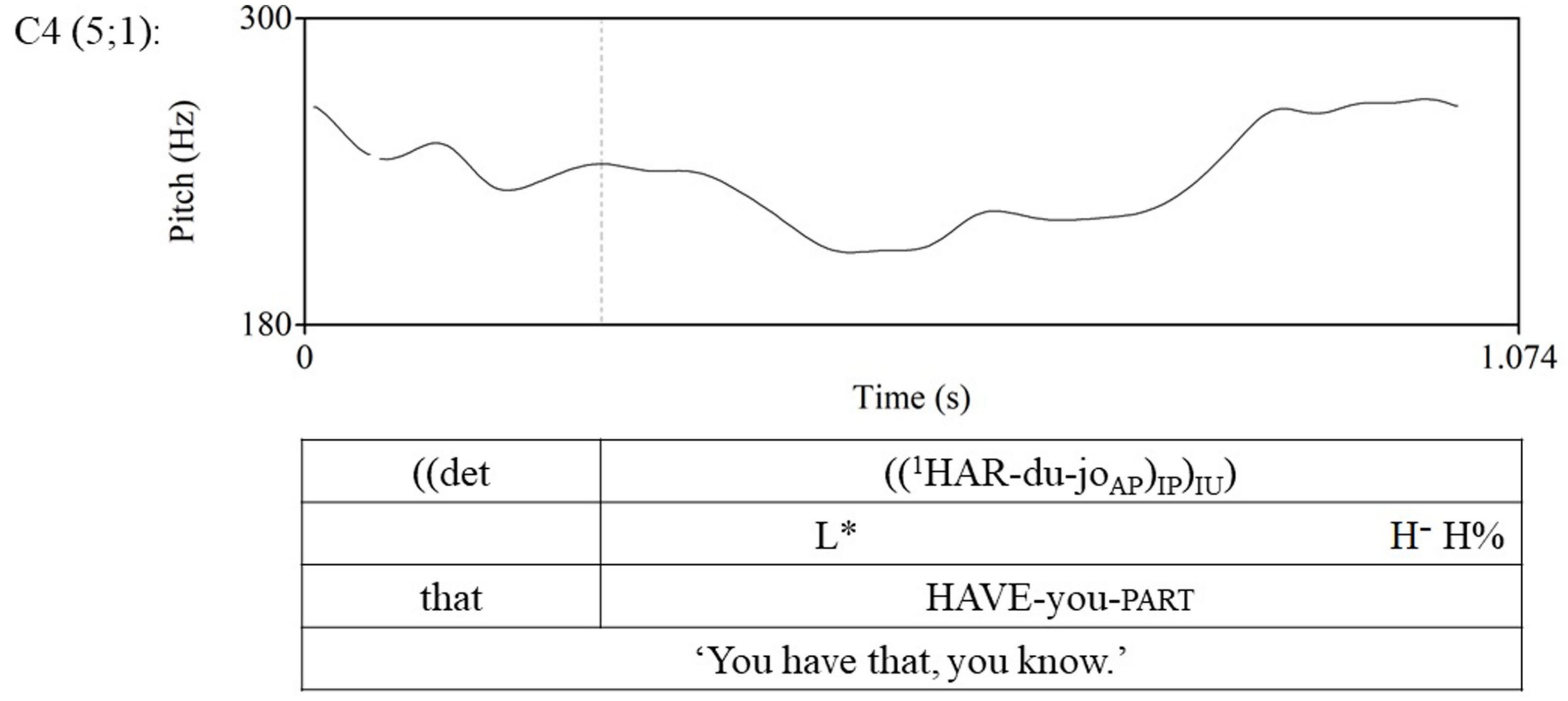
F0-contour of ‘non-PF utterance’ with similar pragmatic effect as a PF utterance.

In the responses from the elicitation task we also find some non-PF utterances (i.e., utterances that do not meet the intonational criteria for PF of having both a focally accentuated polarity carrier and an additional succeeding accentuation) that seem to have a pragmatic effect similar to a PF uttearnce. Consider the example in Figure 8 from one of the participants produced in the Inf-Bel condition after the handpuppet had said *I wish I had something to play with while taking a bath!*

The utterance in Figure 8 has a focally accentuated finite verb, but since there is no following accentuation later in the utterance, it is not considered PF utterance. However, a sentence-internal *jo* follows as an unaccented syllable in the tail of the rising tone. The utterance seems to have a similar effect as a PF response would have had: the participant signals that there is some sort of conflict between the handpuppet’s belief (that he does not have any toys to play with in the bath) and the current state of affairs (that he owns rubber ducks).

## 4. Discussion

The aim of our study was to investigate whether Norwegian-speaking children aged two to five years can produce intonation utterances realized with ‘Polarity Focus’ (PF), and if so, whether we would find a gradual development of their productions in contexts of increasing complexity. While we found productions of PF in all age groups tested and only in felicitous contexts, our hypothesis of a gradual development was only partially supported by our data.

Overall, our findings show that children produce PF from as early as age two. We take this to suggest that they are also (at some level) able to evaluate the truth or falsity of a proposition and attribute a contextually available proposition to their interlocutor from around this age. Furthermore, our findings show that young children can express their affirmation or denial of this truth-conditional content by intonational means. Already from the age of two and onwards, children seem to use intonation naturally and efficiently as a communicative device and in this case specifically to signal epistemic vigilance toward an attributed propositional content. This is also likely to involve an intention to modify their interlocutor’s epistemic state.

As expected, the ability to use PF to express the denial of an inferred false belief seems to arise around four years of age. However, the percentage of PF productions in this condition was overall low (7%). Since this is the most complex condition of our design and only expected to be mastered by the older children, the low percentage of PF productions was not surprising. Our findings align with previous findings in the Theory of Mind literature where the ability to linguistically express an understanding of others’ false beliefs manifests around four years of age (*cf.* Wellman et al., 2001).

Our finding that PF was produced by two-year-olds in both the Pos-Aff, the Pos-Den and the Neg-Den conditions support previous research that show that children are able to both reject false and accept true statements before their second birthday (Lyon et al., 2013), and that two-year-olds spontaneously correct assertions they believe to be false (Pea, 1982). In fact, it was the Neg-Den condition that had by far the highest percentage of PF productions in all age groups. This finding is surprising since the literature suggests that an increasing number of negations increases the complexity of the test conditions (*cf.* Just and Carpenter, 1971). Although this may be the case at a general level, we see no evidence of this in our data: Even two-year-olds master the more complex context where they need to contradict a prior negative belief. This result is in line with Pea (1982), who shows that the ability to correct false statements appears prior to their expressing agreement with true statements. Intonation, and in this case PF, seems to offer young children an easily accessible linguistic strategy for communicating their attitude toward a propositional content, enabling even children as young as two years to express this higher-level metarepresentational content without having to verbalize it.

This high percentage of PF productions in the Neg-Den condition suggests that it was the most natural context for PF production in our task. A growing body of research shows that from very early on children monitor the reliability of the information communicated (Gliga and Csibra, 2009; Koenig and Woodward, 2010; Sperber et al., 2010). It is possible that signaling a denial or an opposing opinion might be more socially important than signaling an endorsement. It could also be that PF is more frequently used by adults in contexts like the Neg-Den condition, and therefore possibly more familiar to children. We know from the study by Turco et al. (2014) that adults produced Verum Focus in 70% of the ‘polarity correction’ contexts where the verbal stimuli used was similar to our Neg-Den condition, involving a mismatch in form of a false negative statement about what was depicted in the visual stimuli. Future research should investigate experimentally how Norwegian-speaking adults use PF, focusing on the different contexts for eliciting PF and in what ways they differ.

We further observed that the Neg-Den condition also provided a natural context for responding with the sentence-initial response particle *jo*, often in combination with PF (such as in (11) above). While according to Noveck et al. (2021) the accurate but surprisingly fast *si* response by the four-year-olds in their study suggested that these children did not aim at modifying the epistemic state of their interlocutor when responding with *si*, our results suggest otherwise. First, they indicate that the ability to produce pragmatically felicitous responses using the response particle *jo* could be present as early as two years of age. Given what we know from the developmental literature of children’s ability to evaluate the truth value of propositional content at such early age (Pea, 1982; Lyon et al., 2013), together with some level of perspective taking (O’Madagain and Tomasello, 2021), it seems likely that, if two-and three-year-olds produce *jo* accurately, they have by the age of four developed a pragmatic maturity that goes beyond relying merely on the minimal semantic representation of the particle. Second, children younger than four years showed mastery of PF production and especially in the Neg-Den condition, a context highly similar to the Negative-Si condition used by Noveck et al. (2021) to elicit the response particle. Furthermore, in our study, children younger than four years spontaneously and felicitously produced the combination of the sentence-initial response particle *jo* and PF. Together, this mastery of both PF and the response particle *jo* at such early age, we believe speak against a limited pragmatic competence involved in the use of this particle at four years of age. To gain a deeper understanding of how the response particle *jo* and PF are related and what the use of them separately and in combination can reveal about children’s developing pragmatic abilities, future research should address this relationship directly, using different approaches and methodologies and a broader set of context types to elicit the two phenomena.

Our data also included participant responses that, although realized without PF, seemed to have a similar pragmatic effect. In the example in Figure 8 above, we discussed how this effect could be due to the presence of a sentence-internal *jo*, which often involves some sort of oppositional feature. Berthelin and Borthen (2019) argue that the procedural meaning encoded by *jo* involves an instruction to the hearer to interpret the proposition expressed as mutually manifest to speaker and hearer, and to use the proposition expressed as a premise for deriving and supporting a contextual implication. As they (2019, p. 25) point out: “*jo* is a useful tool when speakers suspect that the hearer will not accept the information they are communicating.” This is also the case for PF. Just like sentence-internal *jo*, PF can be used when a speaker needs to convince her interlocutor of the epistemic status of the proposition expressed and of the conclusions that can be drawn from it. Future research should also investigate the relationship between the sentence-internal particle *jo* and PF in more detail. If the two phenomena are closely related, in what ways do they differ, and what triggers the use of them in combination?

We have suggested that utterances carrying PF have an affinity with echoic use in the relevance-theoretic sense (Wilson, 2012): PF utterances involve both an attribution of the proposition expressed, and they enable the speaker to convey her attitude to this proposition in the form of a denial or an affirmation. Although this is a rather simple form of echoic use, the attitude being explicitly conveyed, our study has shown that children master such echoic uses from a very early age. This has potential implications for theoretical accounts of the development of other, more complex forms of echoic use such as verbal irony, which is characterized by the speaker tacitly echoing and expressing a dismissive attitude to an attributed thought (Wilson, 2012). These uses have been shown to have a protracted development (Falkum and Köder, 2020) with some comprehension abilities emerging around the age of six years. Our results show that the ability to express an endorsing or dismissive attitude to an attributed thought (expressed explicitly in the context in the simplest cases) emerges much earlier. In this way, intonational competence, and more specifically the ability to use PF appropriately, could be seen as a precursor to ironical uses.

Finally, we would like to mention some caveats. First, we have claimed that PF production involves attribution of the thought that is affirmed or denied by the use of PF. However, the design of the verbal stimuli in our first three PF conditions (Pos-Aff, Pos-Den, Neg-Den) makes it difficult to tease apart this ability from the ability to metarepresent a thought (without having to attribute it), since the handpuppet explicitly expresses his prior beliefs. There is solid evidence that four-and five-year-olds can attribute thoughts. One possibility then is that the two-and three-year-olds do not attribute the thought they are affirming or denying, but are merely metarepresenting a contextually available thought. However, this analysis leaves open the question why a speaker would produce PF in the first place: The informative intention of a speaker who uses PF is to convey her affirmation or denial of a metarepresented thought. Why would she convey this if not to modify her interlocutor’s epistemic state (which does involve thought attribution)? The production of PF does not make sense in a context where the speaker merely metarepresents the thought without attributing it to someone. If two-and three-year-olds did not have this ability (and as a consequence they are not aiming at modifying their interlocutor’s epistemic state), we should expect them to produce less PF overall than the older children, simply because they would not experience relevant situations for the production of PF. However, we find no significant differences in PF productions between the age groups in our study. It seems likely, therefore, that the ability to attribute thoughts is also involved in the felicitous production of PF, and that this ability is present already from the age of two years.

Second, our experimental setting posed some challenges especially for the youngest participants. Although the experimenter made an effort to make the conversation as natural as possible, some of the two-year-olds had trouble adapting to the experimental setting or did not feel familiar enough with the experimenter (even though all participants who wanted to bring a familiar caretaker were given the opportunity to do so), and refused to speak. This could have masked their intonational competence. Finally, the use of production data as a source of evidence for pragmatic competence requires an interpretation of children’s communicative intention, which is inevitably speculative (Zufferey, 2020). Moreover, production data are often thought to underestimate children’s performance compared to comprehension data. However, since the conditions in our structured elicitation task are specifically designed to elicit PF, less is left to speculation compared to spontaneous productions in unstructured contexts. We also believe that for the study of early pragmatic development, production data provide a valuable source of insight, especially because controlled comprehension experiments may not be feasible with children in the youngest age groups. However, our conclusions in this paper inevitably rest on our interpretation of the production data.

## 5. Conclusion

Our study provides the first experimental evidence that Norwegian-speaking children are able to produce intonation utterances realized with ‘Polarity Focus’ from an early age. We suggest that the mastery of the production of PF, as well as their ability to produce the *jo* particles in appropriate contexts, can be seen as an early linguistic manifestation of the cognitive abilities for the attribution of thoughts and epistemic vigilance toward propositional content.

At a more general level, our study provides insight into the role of intonation as part of a broader pragmatic competence. An overarching aim was to start filling a gap in the literature by combining suprasegmental phonology and cognitive pragmatic theory. We provide experimental evidence for the pragmatic functions of intonation, which in the case of PF allows speakers to communicate a positive or negative attitude toward a metarepresented proposition. Our exploratory analyses of the *jo* particles also contribute some insight into children’s developing metarepresentational abilities.

We believe to have shown that studying intonational production can be useful as a window into children’s pragmatic competence. Although our results did not fully support the developmental hypothesis, they provide evidence of the intonational productions of children aged two to five years and a piece of information about their developing pragmatic competence which is currently missing in the literature. We hope to see many more studies of children’s intonational competence during this crucial developmental period in the coming years.

## Data availability statement

The raw data supporting the conclusions of this article will be made available by the authors, without undue reservation.

## Ethics statement

The studies involving human participants were reviewed and approved by NSD - Norwegian Center for Research Data. Written informed consent to participate in this study was provided by the participants’ legal guardian/next of kin.

## Author contributions

LH: conceptualization, experimental design, data collection, coding of the data, writing–original draft preparation, writing–review and editing, visualization, data analysis, and project administration. IF: conceptualization, experimental design, writing–review and editing, data analysis, and supervision. All authors contributed to the article and approved the submitted version.

## Conflict of interest

The authors declare that the research was conducted in the absence of any commercial or financial relationships that could be construed as a potential conflict of interest.

## Publisher’s note

All claims expressed in this article are solely those of the authors and do not necessarily represent those of their affiliated organizations, or those of the publisher, the editors and the reviewers. Any product that may be evaluated in this article, or claim that may be made by its manufacturer, is not guaranteed or endorsed by the publisher.

## Footnotes

^1^The utterance is transcribed using the parentheses notation convention of the Trondheim model (e.g., Nilsen, 1992; Fretheim, 2002), a framework developed for analysis of Norwegian intonation. The line below the transcription contains an annotation of the utterance’s realized tones, and the two last lines are the English translation of the utterance. Capital letters indicate a focal accentuation characterized by an initial realization of a lexical word tone marked by a parenthesis-initial ^1^ for Accent 1 (L*) or ^2^ for Accent 2 (H*L), followed by a tonal rise to an extra high tone (H^−^) marked by a parenthesis-final _AP_ followed by an _IP_. Norwegian utterances must have at least one, and never more than two, focal accentuations. This tonal movement, starting with the onset of a word tone and then rising from the L(ow) tone to an extra high – focal – tone (H^−^), is the most important information structural feature of (East) Norwegian intonation. Focal accentuations signal which part of the utterance is to be interpreted as new information, and which part constitutes information that provides the hearer with relevant context to interpret the new information against (e.g., Fretheim, 2002). Non-focal accentuation is also realized with an initial lexical word tone (^1^ or ^2^) and is followed by a tonal rise to a high, but not extra high, tone ((H)), marked in the transcription by a parenthesis-final _AP_. Unaccented segments have no lexical word tone and are separated from accented words either by an opening parenthesis to the right or by a dash (−) to the left in the transcriptions.

^2^Note that the response particle (i.e., the sentence-initial) *jo*, and the pragmatic particle (i.e., the sentence-internal) *jo* have different etymologies (*cf.* Berthelin and Borthen, 2019, pp. 4, 27fn2).

^3^Note, however, that whereas the relevance of *jo* is limited to preceding contexts containing a negative surface structure, PF is possible as a response to both negative and positive surface structures.

^4^Cf. Blakemore (1987, 2002) and Wilson (2011, 2016) for more on the distinction between ‘conceptual meaning’ and ‘procedural meaning’.

^5^See also Turco et al. (2013) for a study comparing German and French realizations of Verum Focus accent in adult speakers.

^6^South-East Norwegian is a dialect spoken in the South-Eastern region of Norway.

^7^Noveck et al. (2021, p. 22) point out that in classic tasks where participants are given statements (e.g., *A robin is a bird*) and the options True versus False, reaction times typically increase in correlation with increasing number of negations (in the answer or the question).

^8^The GLMM model that included Subjects as a random factor did not converge.
